# Temporal dynamics of muscle, spinal and cortical excitability and their association with kinematics during three minutes of maximal-rate finger tapping

**DOI:** 10.1038/s41598-020-60043-0

**Published:** 2020-02-21

**Authors:** Elena Madinabeitia-Mancebo, Antonio Madrid, Amalia Jácome, Javier Cudeiro, Pablo Arias

**Affiliations:** 1 Universidade da Coruña, Neuroscience and Motor Control Group (NEUROcom); Department of Sport and Physical Education; Department of Physiotherapy, Medicine and Biomedical Sciences; Institute of Biomedical Research of A Coruña, A Coruña, Spain; 2 Universidade da Coruña, MODES Research group, CITIC, Department of Mathematics, A Coruña, Spain; 3 Centro de Estimulación Cerebral de Galicia, A Coruña, Spain

**Keywords:** Motor cortex, Spinal cord

## Abstract

We tested peripheral, spinal and cortical excitability during 3 minutes of unresisted finger tapping at the maximal possible rate, which induced fatigue. Subsequently, we studied the temporal dynamics of muscle fatigue, expressed in the tapping movement profile, and its relationship to neural systems using mixed model analyses. The tapping rate decreased by 40% over the duration of the task. The change in the amplitude of the range of motion was not significant. The excitability of the flexor and extensor muscles of the index finger was tested via evoked potentials obtained with various types of stimulation at various levels of the motor system. The change in spinal excitability with time was evaluated considering the simultaneous changes in muscle excitability; we also considered how spinal excitability changed over time to evaluate cortical excitability. Excitability in the flexor and extensor muscles at the different levels tested changed significantly, but similar excitability levels were observed at notably different tapping rates. Our results showed that only 33% of the decrease in the tapping rate was explained by changes in the excitability of the structures tested in the present work.

## Introduction

Determining the central mechanisms involved in muscle fatigue is important from a physiological perspective and can also have relevant implications from an applied perspective when we refer to sports, ergonomics or certain pathological conditions.

These mechanisms have been studied thoroughly in the case of isometric muscle contractions; they include changes in excitability both at the spinal cord and M1 networks^1–8^.

Another type of muscle activity corresponding to the contractions performed during rhythmic repetitive movements (RRMs) is essential in daily living and may result in fatigue. Traditionally, their central expressions of fatigue have been studied at the point at which an activity has been completed^9,10^, which is a limitation because the CNS recovers very quickly when the activity ends^11,12^. Several works recently tested fatigue at the central level immediately following the end of unresisted RRMs without allowing time for CNS recovery^5,13,14^. The reduction in the maximal movement rate was greater after 30 s of finger tapping (*ft*) than that after 10 s, and the reduction was accompanied by an increase in the excitability of M1 GABA_*b*_ interneurons, which was more pronounced after 30 s of performing the task^5,13^. Interestingly, spinal excitability, which was tested by measuring cervicomedullary evoked potentials (CMEPs), increased during the waning of the tapping rate^14^, which is essentially different from the outcome when fatigue is caused by isometric exercise performed for the same duration and executed with the same body segment^14^.

However, the description of how corticomuscular excitability changes with RRM fatigue development has not been performed, as previously done for isometric activities^15–17^. Another un-resolved point is whether the changes in excitability along fatiguing RRMs are explicative for the changes observed at the kinematics of the movement.

In this work, we have evaluated the temporal-dynamic changes in muscle fatigue during maximal rate finger tapping. We recorded motor evoked potentials (MEPs) “in hand muscles induced” by single-pulse transcranial magnetic stimulation (TMS) over the contralateral M1. We also conditioned this MEP (MEPc) using a previous pulse (*paired-pulse* TMS). In another session in which the same subjects participated, CMEPs were induced by single-pulse TMS at the cervicomedullary junction level, and compound muscle action potentials (CMAPs) were obtained by percutaneous electrical stimulation of Erb’s point. Potentials were acquired while the subjects performed 3 minutes of *ft* at the maximum possible rate. Each subject completed two sessions, and the changes in *ft* during the task in the two sessions were compared.

Based on previous studies employing much shorter task durations^5,14^, our hypothesis is that the *ft* rate will decrease very rapidly during the first few seconds of the task; then, with task progression, the decrease in the *ft* rate should attenuate. The *ft* movement amplitude will change more shallowly^5,13,14^. We previously demonstrated a rapid decrease in the *ft* rate at 30 s and showed that this decrease occurred with increased levels of spinal excitability. Therefore, we do not expect that a reduction in spinal excitability produces the decrease in the tapping rate along the task; rather, we hypothesized that the key mechanism is likely increased inhibition or lack of facilitation at supraspinal levels.

## Methods

### Participants

Ten of 14 healthy participants completed the two scheduled sessions (5 women; age range [19-42 yrs]; all right handed). The four participants who withdrew from the study refused to participate in the session that included CMAP and CMEP testing due to the discomfort produced by these types of stimulation. The data obtained from these participants were not included in the data analyses. The two sessions, which occurred in a randomized order across the participants, were identical except for the type of stimulation received. None of the participants took drugs or performed strenuous physical labour during the week prior to the experimental sessions. The study was approved by the Ethics Committee of the University of A Coruña. The methods used in this study conformed to the principles set forth in the Declaration of Helsinki, and all of the participants signed informed consent forms.

### Protocol

The participants executed *ft* at their maximal possible rate from the very beginning to the end of the 3-min task while receiving verbal encouragement. The instructions given to the subjects were “*Tap with the index finger at your maximal possible rate from the very beginning and along the whole task, which lasts 3* *min*”. No instructions regarding the range of motion (ROM) amplitude of the movement were provided.

**Figure 1:**
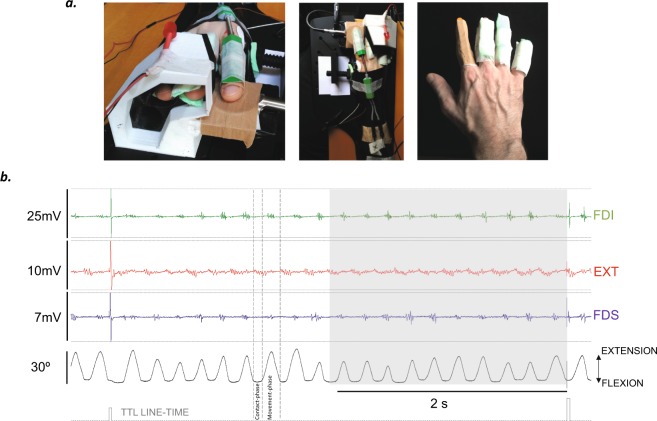
(**a**) The 3D fixation system and hand preparation for the experiments. (**b**) An example of recordings (CMAP-CMEP session); the shaded area corresponds to the 2 s prior to the trigger, and kinematics were computed throughout the 3-min task in this time-window. The activities of the three recorded muscles (the FDI, EXT, and FDS) are shown, as well as the goniometric recording (in black) and the pulses of the TTL triggers (grey).

Each participant’s dominant hand was firmly but comfortably fixed to a 3D hand-fixation structure with the thumb in abduction (Fig. 1a)^13^. *Ft* was executed on a force sensor (Biometrics P200, UK) while index finger movement was monitored with an electrogoniometer (Biometrics S100). A Biometrics K800 unit amplified the signals from both sensors and sent them to a CED1401 (unit-1). Signal 6.0 software (Cambridge, UK) sampled the recordings at 10 KHz and stored them in a computer for off-line analysis.

The signal from the goniometer was also sent to another CED1401 (unit-2, sampling rate 100 KHz) controlled by Sequencer (Cambridge, UK). The sequencer used an algorithm to trigger stimulation at specific points in the tapping cycle (see below for further details). These triggers were also sent to the CED1401 unit-1 to determine the timing of stimulation for posterior signal analysis.

Surface EMG recordings were acquired with a Digitimer D360 (bandpass 3–3000 Hz, gain x250) and sent to CE1401 unit-1. Three different hand muscles were monitored: the *first dorsal interosseous*, *extensor indicis*, and *flexor digitorum superficialis* (FDI, EXT, and FDS, respectively) muscles. The FDI and EXT are the specific flexor and extensor muscles, respectively, of the index finger. Prior to protocol execution, the subjects performed active extension of the index metacarpophalangeal joint to determine their maximal active ROM.

### Stimulation

We evaluated neuromuscular transmission and muscle excitability via CMAPs by stimulating Erb’s point (Digitimer DA7AH stimulator; pulse width 200 µsec). The cathode was placed on Erb’s point in the supraclavicular fossa, and the anode was placed over the acromion. At rest before *ft*, the stimulus intensity was increased stepwise to determine the intensity at which the M-waves stopped increasing in all the tested muscles. During the task, we used an intensity 50% greater than the supramaximal intensity for stimulation. The intensities used ranged from 75 to 160 mA.

Stimulation of corticospinal axons with a Magstim 200^2^ magnetic stimulator connected to a 110 double-cone coil was used to test spinal excitability. The centre of the coil was placed over the inion (slightly lateral in some subjects), and the direction of the monophasic-pulse current flowed down in the coil. The stimulus intensity was adjusted to induce CMEPs ≈5–10% of the CMAP size obtained at rest prior to execution of the protocol (Table 1).

**Table 1:** Peak-to-peak amplitudes and RMS values of the potentials at baseline (the mean and standard error of the mean (SEM)) across participants.

		CMAP		CMEP		MEP		MEPc	
		Amplitude	Rms	Amplitude	Rms	Amplitude	Rms	Amplitude	Rms
**FDI (mV)**	**MEAN**	**18.288**	**4.216**	**0.874**	**0.263**	**1.937**	**0.476**	**1.382**	**0.345**
	*SEM*	*1.383*	*0.334*	*0.406*	*0.101*	*0.438*	*0.100*	*0.372*	*0.085*
**EXT (mV)**	**MEAN**	**10.521**	**2.923**	**0.519**	**0.211**	**0.826**	**0.252**	**0.617**	**0.204**
	*SEM*	*1.226*	*0.348*	*0.155*	*0.041*	*0.222*	*0.053*	*0.188*	*0.041*
**FDS (mV)**	**MEAN**	**7.753**	**1.963**	**0.432**	**0.164**	**0.311**	**0.133**	**0.264**	**0.122**
	*SEM*	*1.053*	*0.320*	*0.109*	*0.021*	*0.048*	*0.010*	*0.026*	*0.004*

Corticospinal excitability was tested by measuring the MEP amplitude induced by single-pulse TMS over M1 with a MagPro X100 with the MagOption and monophasic pulses. Postero-anterior currents were induced in the brain by a figure-eight coil (MC-B70) placed over the hot-spot of the FDI muscle. The hot-spot was defined as the coil position at which the largest and more consistent MEPs were evoked at rest on the FDI muscle, before the fatiguing protocol. This was tested by delivering single TMS pulses at suprathreshold intensity. This position on the scalp was marked with a pen for monitoring coil placement during the session. For testing excitability during FT, the stimulus strength was adjusted to achieve MEPs ≈5–10% of the CMAP size obtained at rest prior to execution of the protocol. A paired-pulse paradigm was also used to assess short intracortical inhibition. With the same system, we tested M1 short intracortical inhibition using a subthreshold conditioning stimulus preceding (by 2 ms) the aforementioned single-pulse MEP. This inter-stimulus interval is suggested to reflect GABA_*a*_ synaptic excitability, whereas shorter ISI are more likely bound to tonic extrasynaptic GABA activity^18^. At rest prior to execution of the protocol, the intensity of the conditioning stimulus was set to produce MEPc values ≈75% of the unconditioned MEP; the Table 2 shows these scores in % of the stimulator output and relative to the active and resting motor thresholds –AMT and RMT. AMT and RMT were determined based on standard procedures^19^, before task execution. Setting this level of inhibition prevents floor effects and enables detection of increases or decreases in inhibition in M1 during the task; these changes in inhibition are key mechanisms related to fatigue^5,13,14,17^.

### Timing of stimulation

Recorded potentials were acquired in two different sessions. One session tested CMAPs and CMEPs, and the other session tested MEPs and MEPc values; the order of the sessions was counterbalanced across participants. During individual sessions, the two types of recorded signals were alternated in their acquisition.

We began each session by recording 10 potentials of each type at rest (*baseline*). The participants were then prompted by an auditory cue to begin *ft*.

During execution of the *ft* task, the stimulation triggers were locked to the tapping cycle. To accomplish this, CED-Sequencer software analysed the signal from the goniometer, and stimulation was applied during the contact phase of the tapping cycle (each tapping cycle had a contact phase and a movement phase; Fig. 1b), which was carried out by analysing consecutive time-bins of 24.6 ms. The average value in each bin (the signal was sampled at 100 KHz) was compared to the value in the previous bin, and the slope was determined. Two consecutive conditions were required to trigger stimulation. The first condition was a score (a difference between consecutive bins) <−6000 units of a 32-bit integer, which is equivalent to a slope of −1.16 mV/sec and determines the point of change from extension to flexion during finger movement (when the finger begins to move downward). The second condition was set to >−1 unit of a 32-bit integer, which is equivalent to a slope of −2.33^−9^ V/sec (≈0) and determines when the finger stops moving (the start of the contact phase). When the sequence of the first and second conditions was detected, stimulation was delivered.

Thus, stimulation was applied in all cases with the finger in the same relative position of the tapping cycle during the stationary phase when the angular velocity is zero. After one stimulus was delivered, the sequencer stopped calculating slopes from the goniometer for 4 seconds to restrict the frequency of stimulation, which can modulate excitability. At the end of the four-second period, the sequencer re-started the calculation as described above and subsequently applied stimulation again; the second stimulation always involved a different modality. Notably, however, the start of the contact phase did not occur immediately after the 4-s time lag. For this reason and because we had programmed 45 triggers during the protocol, the duration of the task was slightly longer than three minutes.

In the MEP-MEPc sessions, the stimulation during *ft* started with MEP testing in half of the subjects and with MEPc testing in the other half of the subjects. Similar alternation in the order of testing was used in the CMAP-CMEP sessions.

### Analysed variables

#### Ft frequency

In a time window of 2 s immediately prior to the TTL (see the grey-shaded area in Fig. 1b), the median frequency of all cycles was calculated from the goniometric recordings. We used customized MATLAB (The MathWorks, Inc) programs to perform these analyses^5,14,20^.

#### *Ft range of movement* (ROM) amplitude

We defined the peak ROM amplitude as the difference in the goniometric recordings between the peak score of the movement phase and the median score of the preceding contact phase for each tapping cycle (Fig. 1b), which was calculated in the same time window as that described above. We considered the median of all cycles as the representative score.

#### *Root mean square* (RMS) of the CMAP, CMEP, MEP, and MEPc values

The RMS was calculated from the areas of the recorded potentials. We used the RMS rather than the peak-to-peak amplitude as the analysed variable because this parameter has been shown to be less dependent on phase cancellation^21,22^.

#### RMS of EMG voluntary activity

We computed the RMS of the EMG background activity during the contact phase, this was the phase at which stimulation was applied. The RMS magnitude of this activity was made relative to the RMS of the CMAP. Customized Matlab programs were used for this purpose.

### Data processing

The rate of *ft* was expressed for each subject as a % of his/her maximal tapping rate during the task regardless of when it was achieved. Similarly, finger-tapping ROM is given as a % of the maximal ROM during the active extension executed before *ft* started.

For the potentials, we first calculated the median of the 10 events acquired at rest prior to execution of the task (*baseline*); the obtained values are shown in Table 1. Next, each potential acquired during the tasks was normalized to the corresponding *baseline* score, which applied to the CMAPs, CMEPs and MEPs but not the MEPc value; the MEPc value during the task was normalized to the unconditioned MEP during *baseline*.

As mentioned above, in session 1, the MEPs and MEPc values were acquired in an alternating manner, as were CMEPs and CMAPs in session 2. Therefore, for the subsequent statistical analyses, we imputed the score of an MEP at an MEPc event time point by calculating the mean score considering the time points immediately before and after that time point. For example, MEP#2 was the mean of MEP#1 and MEP#3; similarly, MEPc#3 was the mean of MEPc#2 and MEPc#4. For the imputation of events #1 and #45 (for which no prior or subsequent events were available to make imputations), event #1 was considered equal to event #2, and event #45 was considered equal to event #44. We proceeded in a similar manner for CMEP and CMAP imputations.

### Statistical analyses

Statistical analyses of the aforementioned variables, as well as of the CMEP scores relative to the CMAP scores (the CMEP/CMAP ratio), were performed, which allowed us to estimate changes in spinal excitability considering the changes that occurred at the same time in muscle excitability and neuromuscular transmission. In the same manner, we calculated the MEP/CMEP and MEPc/MEP ratios. Also, the RMS of the EMG voluntary activity was made relative to the CMAP-RMS.

Linear mixed-effects models were used to describe the *task progression* (in time) of the *ft* rate, the ROM amplitude, and excitability scores. For the excitability scores, we considered the RMS of the potentials (normalized in relation to *baseline*) and the ratios mentioned above. Accordingly, we fitted fourth-order polynomials in which the subjects were modelled as random effects.

Finally, we examined associations between changes in *ft* profiles (the *ft* rate and *ft* ROM amplitude) and changes in excitability over time. Therefore, a linear mixed-effects model was used to explain changes in *ft* frequency and ROM amplitude as a function of time and excitability (CMAP, CMEP/CMAP, MEP/CMEP and MEPc/MEP), with random effects on the subject. “Linear and quadratic functions were explored”:

$${{\rm{Y}}}_{{\rm{i}}}={\alpha }_{0}+{\alpha }_{1}({{\rm{T}}}_{{\rm{i}}})+{\alpha }_{2}{({{\rm{T}}}_{{\rm{i}}})}^{2}+{\beta }_{1}({{\rm{X}}}_{{\rm{i}}})+{\beta }_{2}{({{\rm{X}}}_{{\rm{i}}})}^{2}+{\rm{\varepsilon }}$$

where Y_i_ is the *ft* profile (the frequency or ROM amplitude), X_i_ is the excitability score (CMAP, CMEP/CMAP, MEP/CMEP or MEPc/MEP), T_i_ is the time (TLL event number) in task progression, and ε indicates the residual error of the model.

The normalizing scores for *ft* frequency (i.e., the maximal frequency at any time point in the task) and amplitude (the maximal active ROM before the task) in the two sessions were analysed using Student’s t-test to identify differences between sessions.

Previously to the above-mentioned analyses, the variables compared using a t-test and the residuals of the nonlinear mixed effects models passed the normality tests.

We performed statistical analyses using R software (*nlme* package). Based on previous recommendations^23^, we set p < 0.01 as the level of significance due to the relatively small sample size of our study.

## Results

Table 1 shows the *baseline* potential scores (obtained prior to task execution) used to normalize the scores during task execution. The intensity of TMS (for MEP and CMEP) was selected to obtain potentials of ≈5–10% of the CMAP size in the corresponding muscle; the stimulation parameters in both sessions are showed in Table 2.

**Table 2:** Stimulation intensities used in the different sessions (the mean, standard error of the mean (SEM)) across participants.

	For cmap	For cmep	For tms- test pulse	For tms-condinditioning pulse	Tms amt	Tms rmt	Conditioning TMS pulse	
							% OF AMT	%OF RMT
**MEAN**	**105.0**	**95.0**	**57.6**	**30.6**	**40.5**	**47.1**	**76.3**	**65.2**
*SEM*	*8.1*	*2.1*	*1.6*	*1.3*	*1.6*	*1.5*	*3.7*	*2.5*

CMAP (mA), CMEP and TMS (% of stimulator output).

**Figure 2:**
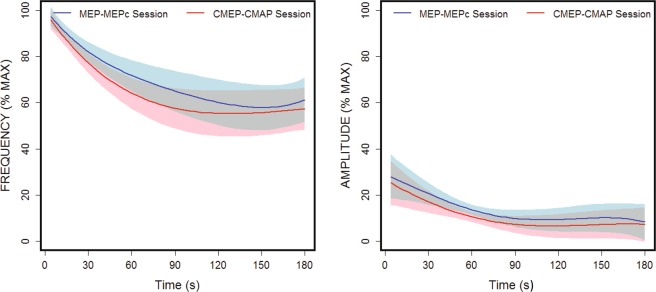
Evolution of the ft frequency (left) and ROM amplitude (right) during the task, which did not differ across sessions (the MEP-MEPc session and CMAP-CMEP session). The blue and pink bands show the 99% CIs.

The maximal active finger index extension ROM (averaged across participants) acquired before execution of the 3-minute task was 50.6° (SEM = 3.3) for session 1 and 51.9° (SEM = 2.3) for session 2; these values were not significantly different (t_9_ = 0.75, p = 0.4) (these scores are equivalent to 100% in the ROM amplitude of the *ft* graphs, Fig. 2). The maximal *ft* frequencies during the task (equivalent to 100% in the frequency of the *ft* graphs, Fig. 2) were 5.7 Hz (SEM = 0.26) and 6.0 Hz (SEM = 0.25) in sessions 1 and 2, respectively; these values were not significantly different (t_9_ = 1.9, p = 0.1).

### Motor behaviour during the task

The 3-min evolution of the *ft* profile did not differ in either frequency (p > 0.2) or ROM amplitude (p > 0.2) in the two sessions. The tapping frequency changed significantly during the task (p < 0.001) in both sessions, decreasing rapidly within the first minute, decreasing more shallowly during the second minute, and reaching a plateau in the third minute (Fig. 2, left panel). The total decrease in the *ft* rate was ≈40%. The change in ROM amplitude during the task followed a similar trend, but the effect was not significant (p = 0.057) (Fig. 2, right panel).

### Excitability during the task

**Figure 3:**
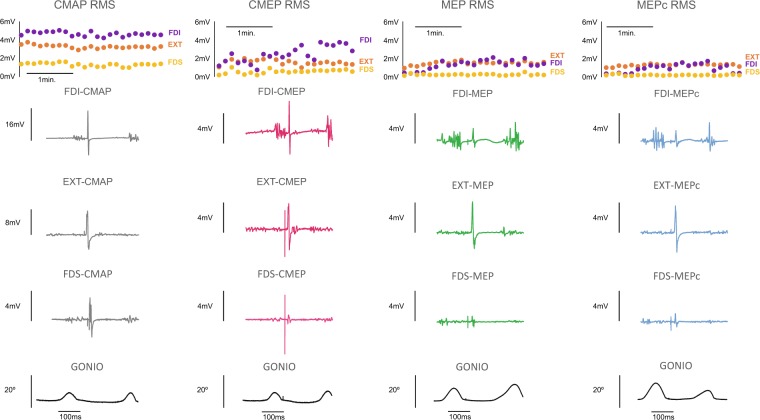
Examples of evoked potentials and goniometric recordings in a participant. The upper section shows the modulation of the different potentials in the three muscles (FDI, EXT and FDS), along task execution.

Figure 3 shows examples of the different potentials and their modulation during task execution in one participant.

#### First dorsal interosseous muscle

**Figure 4:**
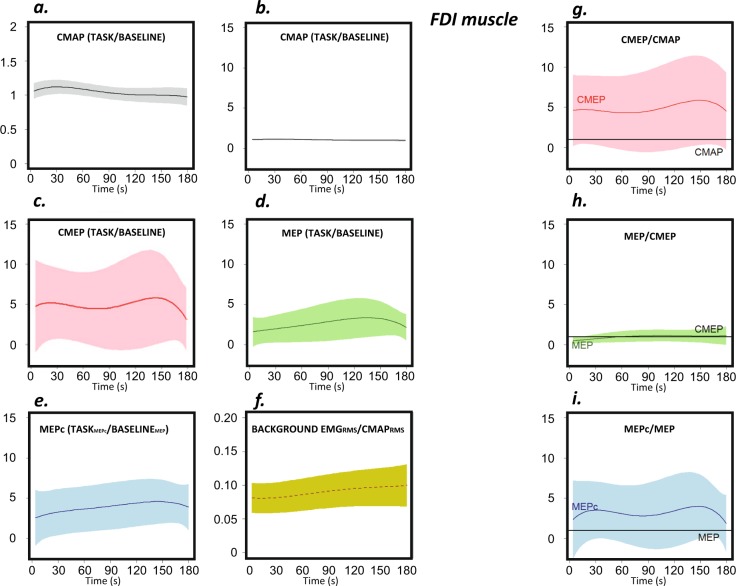
Behaviour of the FDI muscle during the task. The graphs show the RMS values of the CMAP (grey), CMEP (pink), MEP (green) and MEPc (blue) and voluntary background EMG activity (yellow) during the three-minute task. Sections (**a**,**b**) present the same data on different Y-axis scales; (**a**) optimizes the view, and (**b**) conforms to the other sections. The sections on the left (**a**–**e**) represent RMS values that have been normalized to the scores obtained at rest prior to execution of the task (except in the case of the MEPc, which was normalized to the baseline MEP). The RMS of the EMG voluntary activity at the time of stimulation is shown made relative to the RMS of the CMAP (**f**). The baseline scores (Table 1) are equivalent to the units for the Y-axis values. The sections on the right (**g**–**i**) depict the ratios.

The CMAP of the FDI changed with task execution (p < 0.001), increasing and reaching a maximum value at approximately 30 s and then decreasing progressively during the remainder of the 3- min task (Fig. 4a,b).

The CMEP was approximately 5-times larger than the *baseline* (rest) value at the beginning of the task. The profile along the 3-min duration of *ft* is shown in Fig. 4c. Notably, however, the plotted profile is merely illustrative because it does not consider the changes in the CMAPs during the task. Analysis of the CMEP/CMAP ratio (Fig. 4g) indicated that spinal excitability changed with time, and the change approached significance (p = 0.020). The ratio remained relatively stable during the first 100 s of the task but then increased to its maximal level at approximately 160 s of task execution (for the remaining variables, MEP and MEPc, and the remaining muscles, we will report the results in the same manner, and p-values will be provided for the CMAP, CMEP/CMAP, MEP/CMEP and MEPc/MEP variables). Thus, CMEP, MEP, and MEPc values will be provided for descriptive purposes but will not be analysed because analyses of these values rather than their ratios is biased for evaluating spinal, cortical and corticocortical excitability.

Figure 4d shows the profile of the MEP during *ft*, and Fig. 4h considers the changes in the CMEPs during the task. The MEP/CMEP ratio (Fig. 4h) changed significantly with task execution (p < 0.001), increasing during the first half of the task and then remaining stable; however, the magnitude of the change was small.

The MEPc profile is shown in Fig. 4e; the values shown in the profile have been normalized to the unconditioned MEP scores at rest. As expected, the scores at *baseline* (prior to task execution; Table 1) indicate that the MEPc (0.345 mV, SEM = 0.085) was smaller than the MEP (0.476 mV, SEM = 0.100); thus, the conditioning pulse reduced the size of the MEP by approximately 26% at rest. Remarkably, however, the MEPc scores were larger than the MEP scores at all times during task execution, showing that the conditioning pulse, which reduced the MEP at rest, produced facilitation during motor execution. When considering changes in the MEPc with time in relation to the MEP *baseline* scores (Fig. 4e; MEPc-during task/MEP-during *baseline*), the MEPc increased progressively until nearly the end of the task. When this evolution is represented relative to the changes in MEPs during the task (Fig. 4i; MEPc-during task/MEP-during task), the change over time appears less pronounced but is still significant (p < 0.01).

#### Extensor indicis muscle

**Figure 5:**
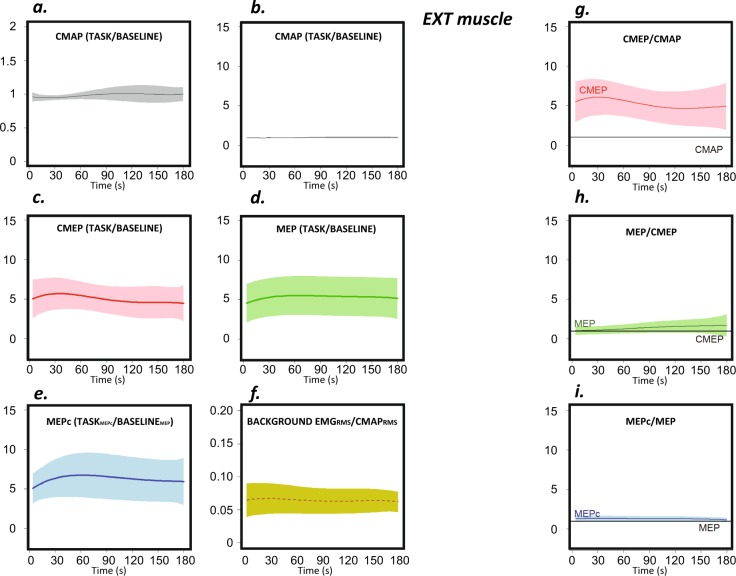
Behaviour of the EXT muscle during the task. The graphs show the RMS values of the CMAP (grey), CMEP (pink), MEP (green) and MEPc (blue) and voluntary background EMG activity (yellow) during the three-minute task. Sections (**a**,**b**) present the same data on different Y-axis scales; (**a**) optimizes the view, and (**b**) conforms to the other sections. The sections on the left (**a**–**e**) represent RMS values that have been normalized to the scores obtained at rest prior to execution of the task (except in the case of the MEPc, which was normalized to the baseline MEP). The RMS of the EMG voluntary activity at the time of stimulation is shown made relative to the RMS of the CMAP (**f**). The baseline scores (Table 1) are equivalent to the units for the Y-axis values. The sections on the right (**g**–**i**) depict the ratios.

For the EXT muscle, the CMAP increased significantly with task progression (p < 0.001), although the magnitude of the change was small (Fig. 5a,b).

The evolution of the CMEPs during the task is shown in Fig. 5c (the CMEP/CMAP ratio in Fig. 5g). Spinal excitability changed significantly with *ft* execution (p < 0.001); when *ft* began, spinal excitability was 5-times greater than the excitability at rest and increased further to reach its peak after 30 s. It then decreased progressively during the 2^nd^ minute and remained stable (approximately 5-times larger than that at rest) during the final minute.

The change in MEPs (Fig. 5d) is shown relative to the change in CMEPs in Fig. 5h. Cortical excitability (the MEP/CMEP ratio) changed as the task progressed (p < 0.001) and reached its maximum value at the end of the task when it was ≈1.5-fold greater than that at the beginning.

The MEPc at *baseline* was smaller than the MEP; the conditioning pulse reduced the RMS by ≈20% (Table 1). Again, as in the case of the FDI, during task execution, the EXT MEPc was larger than the MEP at all times (Fig. 5d,e). As the task progressed, the change in the MEPc/MEP ratio approached statistical significance (p = 0.012; Fig. 5i); the increase was small during the first 30 s of the task and then decreased slightly and progressively.

#### Flexor digitorum superficialis muscle

**Figure 6:**
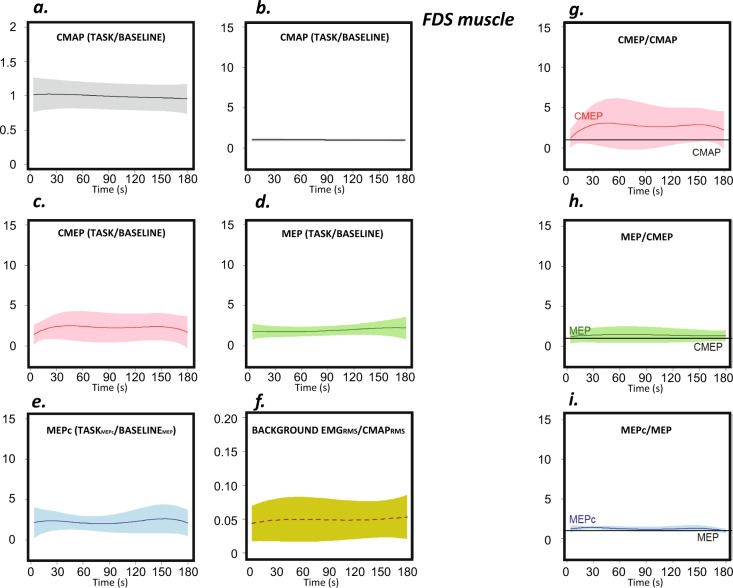
Behaviour of the FDS muscle during the task. The graphs show the RMS values of the CMAP (grey), CMEP (pink), MEP (green) and MEPc (blue) and voluntary background EMG activity (yellow) during the three-minute task. Sections (**a**,**b**) present the same data on different Y-axis scales; (**a**) optimizes the view, and (**b**) conforms to the other sections. The sections on the left (**a–e**) represent RMS values that have been normalized to the scores obtained at rest prior to execution of the task (except in the case of the MEPc, which was normalized to the baseline MEP). The RMS of the EMG voluntary activity at the time of stimulation is shown made relative to the RMS of the CMAP (**f**). The baseline scores (Table 1) are equivalent to the units for the Y-axis values. The sections on the right (**g–i**) depict the ratios.

Although the magnitude of the change in CMAPs was discrete, the CMAPs decreased significantly along the *ft* task (p < 0.001) (Fig. 6a,b).

The profile of spinal excitability (normalized to baseline scores) is shown in Fig. 6c, and the CMEP/CMAP ratio is shown in Fig. 6g. The ratio changed during execution of the task (p < 0.001), increasing for the first 30–40 s and then remaining relatively stable. The changes in the MEPs during the task (Fig. 6d) are shown relative to the changes in the CMEPs in Fig. 6h. The change in the MEP/CMEP ratio with *ft* was small in magnitude but statistically significant (p < 0.001)

The MEPc changes resembled the pattern observed for the other index flexor muscle (the FDI) but were smaller in magnitude. Remarkably, again, the MEPc at rest was smaller than the MEP on average (Table 1), but this pattern reversed with *ft* execution, and the MEPc became larger than the MEP (Fig. 6d,e). The changes in MEPc relative to MEP evolution during *ft* (Fig. 6i) were significant (p < 0.01).

### EMG voluntary activity in the contact phase during the task

Stimulation was applied during the contact phase of the tapping cycle. At this phase the voluntary EMG activity -RMS-, made relative to the changes in the RMS of the CMAP at the same time, changed significantly during task execution for the FDI, EXT, and FDS (p < 0.001, in all cases). However, in the three muscles, the effect was small in magnitude (section *f* of Figs. 4–6).

### Associations between changes in excitability and motor behaviour during the task

**Figure 7:**
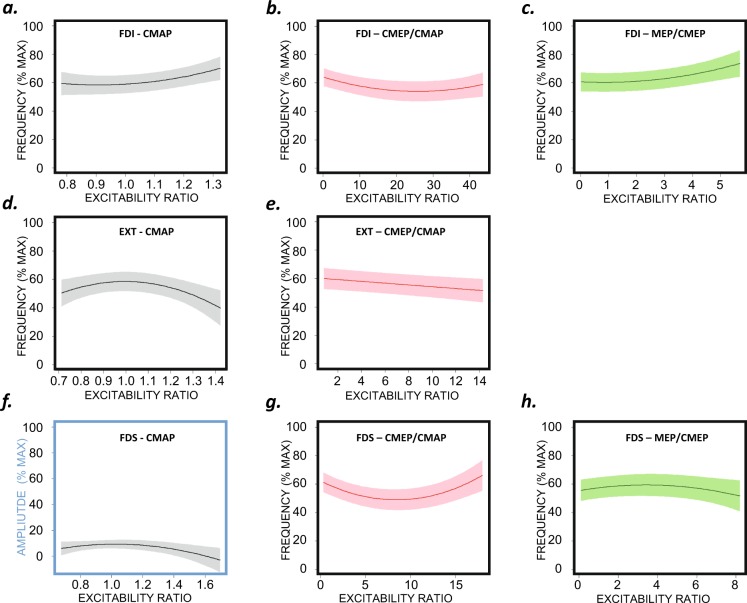
Graphic representation of the functions explaining the association between changes in excitability and motor behaviour (i.e., changes in the ft frequency or amplitude). Only significant relationships are presented. The upper plots (**a–c**) represent the FDI, the middle plots (**d,e**) show the EXT, and the lower plots (**f–h**) show the FDS. The grey, pink and green plots depict muscle, spinal and cortical excitability, respectively. All significant associations were established based on the change in ft frequency except in the case of CMAP excitability tested on the FDS, which explained the observed changes in the ft amplitude (section **f**).

These analyses show whether muscular excitability and neuromuscular transmission (CMAP), spinal excitability (CMEP/CMAP) and cortical excitability (MEP/CMEP and MEPc/MEP) explain changes in *ft* behaviour. The significance of the association is shown in Table 3 (in the quadratic model, significance refers to a significant effect over the linear fit). The estimated models are presented in Table 4, and the significant relationships are plotted in Fig. 7. Notably, in Fig. 7, the X-axis reflects the excitability in each case rather than the time progression. In fact, the greatest excitability may occur at the beginning of the task (see, for instance, Fig. 4a, which shows the CMAP of the FDI) or at any other time.

**Table 3:** Associations between changes in tapping frequency, tapping amplitude and excitability during the 3-min task.

Muscle		Tapping Frequency		Tapping Amplitude	
		Linear	Quadratic	Linear	Quadratic
FDI	CMAP-RMS	**p < 0.001**	**p < 0.01**	N.S	N.S
	CMEP/CMAP-RMS	**p < 0.001**	**p < 0.001**	N.S	N.S
	MEP/CMEP-RMS	**p < 0.001**	**p < 0.01**	N.S	N.S
	MEPc/MEP-RMS	N.S	N.S	N.S	N.S
EXT	CMAP-RMS	N.S	**p < 0.001**	N.S	N.S
	CMEP/CMAP-RMS	**p < 0.001**	N.S	N.S	N.S
	MEP/CMEP-RMS	N.S	N.S	N.S	N.S
	MEPc/MEP-RMS	N.S	N.S	N.S	N.S
FDS	CMAP-RMS	N.S	N.S	N.S	**p < 0.01**
	CMEP/CMAP-RMS	N.S	**p < 0.001**	N.S	N.S
	MEP/CMEP-RMS	N.S	**p < 0.01**	N.S	N.S
	MEPc/MEP-RMS	N.S	N.S	N.S	N.S

**Table 4:** Estimated coefficients in the linear and quadratic models with a significant effect on finger-tapping behaviour.

Muscle		CMAP-RMS		CMEP/CMAP-RMS		MEP/CMEP-RMS	
		Linear	Quadratic	Linear	Quadratic	Linear	Quadratic
FDI		**Tapping Frequency**		**Tapping Frequency**		**Tapping Frequency**	
	α_0_	73.5^***^	148.1^***^	97.5^***^	99.9^***^	95.5^***^	96.5^***^
	α_1_ (T_i_)	−2.31^***^	−2.27^***^	−2.36^***^	−2.38^***^	−2.41^***^	−2.38^***^
	α_2_ (T_i_)^2^	0.036^***^	0.035^***^	0.035^***^	0.036^***^	0.036^***^	0.035^***^
	β_1_ (X_i_)	20.05^***^	−123.8	−0.185^***^	−0.79^***^	1.43^***^	−0.95
	β_2_ (X_i_)^2^		68.2^**^		0.015^***^		0.57^**^
EXT		**Tapping Frequency**		**Tapping Frequency**		**Tapping Frequency**	
	α_0_		−6.30^***^	99.2^***^			
	α_1_ (T_i_)		−2.48^***^	−2.62^***^			
	α_2_ (T_i_)^2^		0.04^***^	0.04^***^			
	β_1_ (X_i_)		203.02^***^	−0.63^***^			
	β_2_ (X_i_)^2^		−102.01^***^				
FDS		**Tapping Amplitude**		**Tapping Frequency**		**Tapping Frequency**	
	α_0_		−0.75^**^		100.1^***^		93.2^***^
	α_1_ (T_i_)		−1.29^***^		−2.59^***^		−2.55^***^
	α_2_ (T_i_)^2^		0.02^***^		0.04^***^		0.04^***^
	β_1_ (X_i_)		56.0^**^		−3.16^***^		2.28^**^
	β_2_ (X_i_)^2^		−27.3^**^		0.187^***^		−0.33^**^

*** p < 0.001, ** p < 0.01; Y_i_ = α_0_ + α_1_ (T_i_) + α_2_ (T_i_)^2^ + β_1_ (X_i_) + β_2_ (X_i_)^2^ + ε

#### Associations between excitability and ft pattern changes

The excitability of the FDI muscle (a flexor muscle) explained the changes in *ft* frequency (Table 3). Figure 7a,c shows that the CMAP and supraspinal excitability (the MEP/CMEP ratio) increased as the tapping rate increased (Table 4). Compared to the linear model, the quadratic model shows a steeper increase in excitability for greater tapping rates.

**Figure 8:**
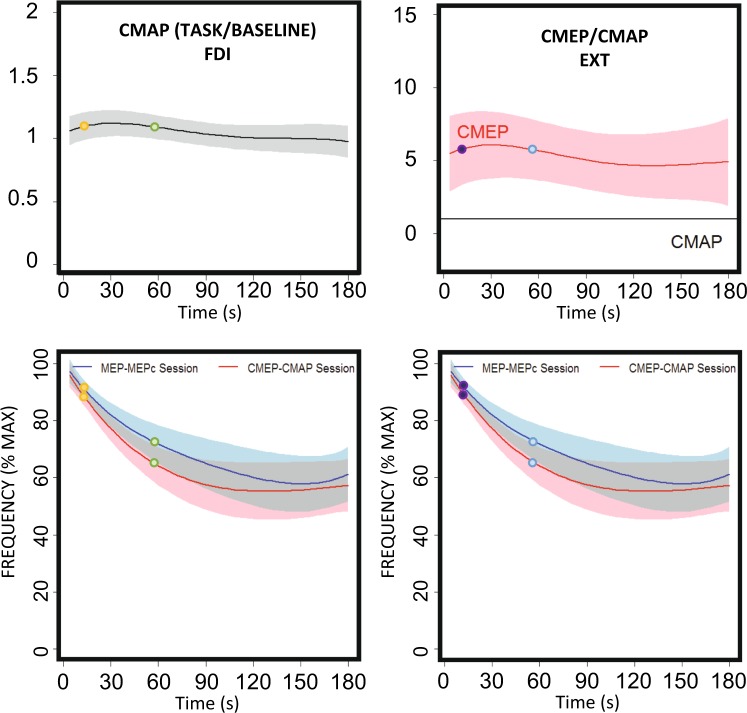
Representation of the poor explicative value of excitability on the tapping rate drop along the fatiguing task. For a same level of excitability (yellow/green and purple/blue dots at the upper plots), the maximal tapping rates were very different (lower plots).

The nature of the relationships depicted in the models is worth considering. For the behaviour of the CMAPs (Fig. 7a), the greatest excitability is associated with a tapping frequency equivalent to approximately 65% of the maximal tapping rate; the reason for this is explained by the CMAP profiles (Fig. 4a, also Table 4) and the tapping rate (Fig. 2, left panel). The fitted models represented in Fig. 4a show that the maximum CMAP score is achieved ≈40 s after *ft* begins. At this time, the *ft* rate was ≈80% of the maximum rate (Fig. 2, left panel). On the other hand, similar CMAP scores can be found both at the very beginning of the task (high tapping frequency) and during the middle and end of the process (lower tapping frequencies) (see Fig. 4a). Therefore, very different *ft* rates occur with very similar levels of excitability (as clearly observed in the schematic representation of Fig. 8), which is why a significant proportion of the change in the frequency of the tapping rate (approximately 40%, see Fig. 2a) is not explained by changes in this excitability variable (a similar pattern is also present for the rest of the variables). According to the fitted quadratic model (Table 4), a change in the *ft* rate of up to 11% is explained by the CMAPs (the predicted *ft* rates vary from 59% for the lowest CMAP score to 70% for the highest level of excitability, Fig. 7a), whereas the global change in the rate was ≈40%.

A similar outcome was observed for the cortical excitability of the FDI (Fig. 7c). The change in FDI excitability explains 13% of the change in the frequency of the tapping rate, corresponding to a difference in *ft* rates between 60% for the lowest values of the MEP/CMEP ratio and 73% for the highest ratio values, whereas the actual decrease in the tapping rate was ≈40%.

The change in spinal excitability (Fig. 7b) explained the change in the *ft* rate (Table 4). However, the relationship was negative, and the reduction in the maximal tapping rate was associated with an increase in the excitability of the motoneurons controlling the FDI muscle.

The changes in excitability recorded in the EXT muscle explained the reduction in *ft* frequency along the 3-minute task, but only in the case of peripheral and spinal excitability (Table 4, Fig. 7d,e). For spinal excitability, the association was linear and negative. Conversely, neuromuscular transmission and muscle excitability (CMAP) displayed quadratic behaviour, showing that higher scores were associated with lower tapping rates.

In contrast to the other muscles, in the case of the FDS, only quadratic functions helped explain changes in the *ft* profiles considering modifications in excitability. The change in the CMAP explained the change in *ft* amplitude (Table 4, Fig. 7f). The changes in spinal and cortical excitability explained the change in the tapping rate (Table 4, Fig. 7g,h).

## Discussion

The first objective of this work was to describe the physiological changes in excitability that occur along the corticomuscular axis during the execution of a simple unresisted repetitive movement performed at the maximal possible rate. The second objective was to determine whether those modifications could explain changes in motor execution.

Fatigue was expressed with a fast drop in the maximal tapping frequency in the first minute of task execution, which was modest in the second minute, and made a plateau in the third part of the task. The R.O.M amplitude showed a similar profile but much milder in magnitude, and the change did not reach statistical significance. At all the tested levels, excitability was modulated significantly along the task, but the changes in excitability explained a modest proportion of the tapping rate drop.

Excitability was tested during task execution and the tapping profiles analysed in time-windows of 2 s. preceding stimulation, therefore avoiding the computation of the few taps disturbed by applying the stimulation. This methodology has been traditionally used to study fatigue during isometric contractions^15–17^; however, for repetitive movements, the typical approach has been to begin evaluating excitability once the task ends^9,10^. We avoided this option because the CNS is known to recover very quickly after muscle contractions that lead to fatigue^11,12^ and because testing excitability at rest may not be adequate to explain the changes that occur during task execution. This last point is supported by the data obtained in the present study and will be discussed later.

The execution of *ft* at the maximal possible rate produced fatigue very early. The instructions given to the subjects were to perform *ft* at the maximal possible rate throughout the duration of the 3-minute task without requirements related to *ft* ROM, and verbal encouragement was provided to them to tap as fast as they could. Under these conditions, fatigue was clearly defined as a waning of the maximal tapping rate: a very rapid decrease was observed in the first minute, a more modest decrease occurred during the second minute, and a fairly constant *ft* rate was achieved during the third minute (approximately 60% of the maximal tapping rate). The *ft* ROM amplitude did not change significantly. At this point, we must consider that during a rhythmic repetitive movement, a close inverse relationship exists between the tapping rate and ROM amplitude. Tapping at the maximal possible rate causes a reduced ROM amplitude; therefore, if participants were to tap with a maximal ROM, their maximal *ft* frequency would be much lower. In this case, the behaviour of the flexor and extensor muscles should probably be different, as would also be the case if the movement were performed against resistance. This is a key point that must be considered to properly understand the scope of our study: the particular relationship between rate and ROM amplitude was observed chiefly because subjects performed maximal-rate *ft* with a very small ROM amplitude (≈25% of the subjects’ maximal ROM amplitude).

The behaviour in the *ft* profile is consistent with that found in previous studies in which similar tasks were evaluated. Performing tapping at the maximum possible rate for 30 s has been shown to reduce movement frequency by approximately 15–20%^5,10,13,14^, as in the present work. The temporal evolution of *ft* ROM showed changes that were less pronounced than the changes in the *ft* rate, which is consistent with previous works that used tapping tasks of much shorter durations^5,13,14^. As previously mentioned, since tapping at the highest rate is bound to produce a smaller ROM, the fatigue related to the tapping rate may not necessary be similarly linked to ROM.

Interestingly, the changes in the excitability of the flexor and extensor muscles during task execution were significant despite the antagonistic functions of these muscles. The extensor works against gravity to lift the finger, and the flexors act to stop extension and perform flexion (Fig. 1b shows that FDI voluntary EMG activity is evident just before peak extension (this activity stops the extension) and just after it (when flexion is accelerated)). These changes in excitability with task progression appeared at different levels of the corticomuscular axis. Importantly, excitability scores acquired through CMEP, MEP and MEPc are dependent on the level of motoneuronal discharge (i.e., EMG background activity) at the time of stimulation. EMG background activity changes with task progression un-avoidably, as it is intrinsic to the execution of a fatiguing task. However, the change of EMG background activity at the time of stimulation was small. Remarkably, the computation the MEP/CMEP, and MEPc/MEP ratios cancels this effect out, but not in the case of the CMEP/CMAP ratio.

Focussing on evoked potentials, the effects on neuromuscular propagation were remarkable. First, they were small in magnitude but significant. The most impacted muscle was the FDI, for which the CMAPs increased by ≈15% over ≈30 s and then progressively decreased. In the other two muscles, the changes in CMAPs with time were also significant but were less pronounced. Second, this finding indicates that the reading and interpretation of signals evoked at the spinal or cortical levels must necessarily consider changes in excitability at the muscular level and importantly, at the time of fatigue development. We addressed this relevant point by computing the CMEP/CMAP, MEP/CMEP, and MEPc/MEP ratios; the analyses of the raw CMEP, MEP, and MEPc values were not considered (although their profiles are depicted in sections c-e of Figs. 4–6). In a few previous studies, similar approaches have been used to evaluate the neural expressions of muscle fatigue^1,14^.

In contrast to the evolution of the CMAP, spinal excitability (i.e., the CMEP/CMAP ratio) exhibited a large change during task execution. Spinal cord excitability was approximately 5-times larger at the beginning of task execution than that at rest both in a specific index finger extensor (the *extensor indicis*) and in a specific index finger flexor (the *first dorsal interosseous* for which flexion is the essential role when the thumb is fixed in abduction^13,24^, as was the case in this study). Spinal excitability in the *flexor digitorum superficialis* was modulated to a lesser extent during task execution. Notably, this muscle does not act specifically on the index finger but also acts on other fingers. As an aside, in our experimental setup, the wrist was fixed in extension, which may contribute to the inhibition of this muscle at presynaptic and postsynaptic levels within the spinal cord^25^. For this reason, the change in excitability that occurs in this muscle during the task, as well as the association between changes in excitability and the *ft* profile (as discussed below), may be quite different from the changes that occur in the other two explored muscles. In the FDI and EXT, spinal excitability changed significantly with time, but in both cases, it remained well above the level of baseline excitability tested at rest, and the deviation from those elevated levels of excitability was small.

Cortical excitability, as measured by the MEP/CMEP ratio, changed significantly during the task in the three muscles. Notably, the magnitude of this change differed in the three muscles. In the two muscles that act specifically on the index finger (FDI and EXT), excitability increased during the 3-min task. In the FDS, the small but significant increase in excitability reached a maximum at approximately 90 s, and excitability then decreased slowly. In light of previous work that has shown a clear increase in cortical excitability, our results were to some extent unexpected^9,10^. However, some of the earlier reports acquired MEPs after fatiguing repetitive movement and/or by testing MEP without considering the modulation of spinal cord and muscle excitability during testing^9,10^ (in our work and in the case of the spinal cord, this modulation was found to be large). Our protocol controls for these sources of bias. Some other studies in which these limitations were controlled have consistently shown increased excitability of inhibitory interneurons (GABA_*b*_) during fatiguing *ft*^5^. We did not specifically test those circuits in the present work, but the current results suggest that the increased excitability of inhibitory GABA_*b*_ -interneurons in M1^5,13^ is likely compensated for by changes in the excitability of some other neural population, as shown by the results of the MEP/CMEP ratio.

We evaluated some of the possible compensatory circuits using paired-pulse TMS with an ISI of 2 ms, likely testing GABA_*a*_ inhibition in M1^26^. Remarkably, a standard paired-pulse protocol for testing this form of inhibition (i.e., using as conditioning pulse intensity 90% of the AMT^27^) produces a very powerful inhibition on the conditioned MEPs; thus, this methodology might impede the detection of increased levels of inhibition generated by the task. To avoid this, at baseline, we set individualized conditioning pulse intensities producing conditioned MEPs smaller the un-conditioned ones, but larger enough to permit its modulation by the execution of the fatiguing task. Unexpectedly, however, during task execution the effect of the conditioning pulse on the MEP was the opposite: it produced facilitation of M1 in all the tested muscles. We have not identified the physiological mechanism that underlies this observation, but it may involve changes in the excitatory/inhibitory balance of M1 during the execution of the fatiguing task. The parameters that we used at rest were selected to permit evaluation of intracortical GABA_*a*_ inhibition^26^. Interestingly, intracortical excitatory circuits can also be tested using similar ISI^28^. This is the case for short intracortical facilitation (SICF), where a subthreshold pulse (delivered 1–5 ms after a suprathreshold pulse) increases the MEP size, an effect that is mediated by M1 excitatory interneurons^29^. One may be tempted to speculate that in our experiment, a similar mechanism may be operating in which the conditioning pulse arrives at a moment at which altered excitatory/inhibitory balance is produced by the task. On the other hand, although our results cannot explain the mechanism underlying this observation, a very relevant implication of these results is the need to test fatigue expressions at the time of fatigue^1,5,13,14^ as the excitatory/inhibitory balance at that time is quite different from the balance at rest^9,12,30^.

Despite the above-described clear and significant changes in excitability, our study was designed to answer a more relevant question: Do these changes in excitability explain the modifications in tapping rate or amplitude? To answer this question we tested the association between the changes in tapping profiles and excitability. In some muscles and variables only linear functions were significant. Thus, the behaviour was well explained by a lineal fit, the change in the tapping profile is therefore constant with the change in excitability (for instance Fig. 7e). In some other muscles and variables, only quadratic functions were significant (Figs. 7d,g). In those with *β*_*1*_ positive and *β*_*2*_ negative (Table 4 Fig. 7d), this means that there is an optimal level of excitability for behaviour, resulting excitability levels above and below this point in poorer performance. If *β*_*1*_ negative and *β*_*2*_ positive (Fig. 7g), there is an excitability level at which the behaviour was the poorest, and levels of excitability above and below produced better performance. Finally in some cases both linear and quadratic models were significant. This means that the tapping frequency (or ROM amplitude) always increases (or decreases) with excitability, but this change is not constant (Fig. 7c). As a whole, the results clearly showed that such dependence appears to exist, but it is fairly limited. Our results show that very similar levels of excitability are observed at very different *ft* rates (Fig. 8). This necessarily means that some mechanism other than the one explored in this work is responsible for a significant proportion of the waning of finger-tapping frequency observed during this repetitive unresisted task. It is well known that peripheral mechanisms related to muscle contractility, which was not tested in this work, play a major role in fatigue^31^. This has recently been confirmed using a *ft* task performed at the maximal possible rate and was shown to occur even when the duration of the task was short (30 s)^13^; in that case, a slowing of muscle relaxation was evident at the end of the task in the presence of a tapping rate decrease of ≈15%. This mechanism appears to be much less relevant in the case of isometric fatiguing tasks^13^. In the present work, we observed decreases in tapping rate of up to 40% at the end of the task; however, the changes in the cortical excitability (MEP/CMEP) of the FDI muscle, which was the parameter with the greatest explanatory value, accounted for *ft* rate reductions of only up to 13% (note that this is approximately 33% of the total decrease in frequency).

Apart from peripheral elements, central mechanisms can also coexist. At this point, one must keep in mind that our work explored an important but limited number of central circuits with potential relevance to fatigue. Other populations of interneurons (inhibitory or excitatory) may play a role in the development of fatigue. This appears to be the case for inhibitory GABA_*b*_ interneurons^5^. Additionally, a putative role of other supraspinal structures involved in motor control (other than M1) has to be considered during fatigue development^32^.

Alternatively, the presence of central adaptations during fatigue might not have a correlate in excitability changes reflected by the MEP. For instance, the reduction in muscle force and central drive to the muscle might not match changes in the MEP features^13,16^. Following this line of thought, the central mechanism of fatigue during RRM might be linked to the precise sequence of activation of antagonistic muscles^33^. However, as far as the corticomuscular axis is concerned, the excitability of the structures explored in our work appears to have a limited impact on the inability of the subjects to maintain the maximal tapping rate during repetitive movement.

### Study limitations

Several points should be considered limitations of our study. First, the study sample size was small. Four of the 14 initially recruited subjects chose not to take part in the CMAP-CMEP session due to the discomfort produced by these techniques. Thus, only 10 participants completed the two sessions. Although similar sample sizes have been used in relevant studies in the field^1,16^, the use of larger samples would allow a more confident estimation of the findings. To account for this limitation, we adopted a conservative approach in our data analyses, and the level of significance was set at p < 0.01, as has been recently recommended^23^.

Second, the extension phase of *ft* in our study was anti-gravitational, and the flexor muscles worked in favour of gravity. Since the movement was not resisted and the mass of the index finger is small (the goniometer is also very light), we have discussed our results as stemming from an unresisted movement, but it is true that the resistance for the extensors and the flexors in our task is not exactly the same. This is important since repetitive movements against higher levels of resistance might present different evolution of muscle excitability and processes related to muscle force production^34^.

Finally, during the recording of the MEP and MEPc, we focussed on the cortical *hot-spot* of the FDI, which has a main role in this task^5,13,14,24^, and with this *hot-spot* we recorded potentials in other two muscles. We adopted this approach since the cortical spatial distribution of neurons targeting hand muscles is overlapped. This has been shown previously by different image studies in human^35,36^ and with neuronal recordings in monkey^37^. Therefore, in our study, even though we were on the hot-spot of the FDI, we were expecting to stimulate a large proportion of the neuronal pool targeting the other two muscles engaged in digital movements. In fact, as observed in our work (Table 1), the MEP-RMS scores for the different muscles represented a similar proportion of their CMAP-RMS. This suggests we tested a similar proportion of the total neuronal pool in the three muscles, during TMS. For such reason, we believe the use of a single *hot-spot* is suitable for our purpose.

## Conclusions

Our work shows that changes in excitability along the corticomuscular axis play a role in the fatigue that develops during unresisted repetitive movements but cannot fully explain the observed decrease in the tapping rate. When muscles that act specifically on the index finger were tested, increased spinal excitability in both the flexor and the extensor was associated with a lower tapping rate. The cortical excitability of the specific flexor muscle tested, which plays a main role in the rapid change from extension to flexion that is necessary to produce higher tapping rates, increased with higher tapping rates.

## Data Availability

The datasets generated during and/or analysed during the current study are available from the corresponding author on reasonable request.
